# Physiological, nutritional, and productive responses of broilers supplemented with lemon, fenugreek, and sesame oils as feed additives

**DOI:** 10.1038/s41598-025-09252-z

**Published:** 2025-07-10

**Authors:** Mohamed A. Fawaz, Ayman A. M. Abd El-Hafez, Yousef A. Abdelmoati, Abdalla H. H. Ali, Hamdy A. Hassan

**Affiliations:** 1 Department of Animal and Poultry Production, Faculty of Agriculture, South Valley University, Qena, 83523 Egypt; 2 Department of Animal Production, Faculty of Agriculture, Al-Azhar University, Assiut, 71524 Egypt

**Keywords:** Lemon, Fenugreek, Sesame oils, Digestibility, Performance, Microbial, Animal physiology, Microbiology

## Abstract

This study objective is to evaluate the influence of lemon, sesame, fenugreek essential oils and their mixture as feed additive on the growth performance, carcass criteria, nutrient digestibility, cecum microbial and certain blood parameters of broiler chicks. A total of 240 one day-old unsexed broiler chickens Ross 308 were divided into 5 treatment groups, each treatment had 6 replicates with 8 birds per each. the birds were fed a basal diet without supplementation (control group), basal diet supplemented with lemon essential oil (LEO) at 400 mg/kg (LEO group), basal diet supplemented with sesame essential oil (SEO) at 400 mg/kg (SEO group), basal diet supplemented with fenugreek essential oil (FEO) at 400 mg/kg (FEO group), basal diet supplemented with combination of LEO, SEO and FEO (Mix group), for 5 weeks. The results revealed that FEO supplementation significantly (*P* < 0.05) improved body weight (BW), body weight gain (BWG), feed intake (FI) and feed conversion ratio (FCR) compared to the control group. At the first week FI was lower in Mix group as well as improving FCR than the control and LEO groups (*P* < 0.05). Datary containing FEO, LEO, and SEO either alone or in combination significantly (*P* < 0.01) improved serum levels of total protein, globulin and urea and crude protein digestibility, increased cecal counts of *Lactobacillus* as well as reducing counts of cecal bacteria, *E. coli,* and *Salmonella.* Therefore, it was found that, in comparison to other treatments, broiler chicks provided 400 mg/kg of fenugreek oil demonstrated improved growth performance and general health condition.

## Introduction

Administering in-feed antibiotics to increase poultry productivity while lowering bird mortality is a typical nutritional approach. Because of worries about possible bacterial multiple resistance^1^, and their detrimental effects on the health of the customers antibiotics were prohibited by the European Union^2,3^. The use of feed additives as an alternative to antibiotics, which are a crucial and influential component of the composition of feed in order to improve the overall health and performance of the poultry, is of significant interest to nutritionists^4^. There has been a lot of interest in investigating products made from aromatic pharmaceutical herbs extract, essential oils and spices as immune-boosting and antibiotic substitutes^5–7^. Numerous beneficial chemicals found in medicinal herbs have been shown to play a significant role as antioxidants, antimicrobials, anti-inflammatory, anti-coccidial properties and enzyme synthesis stimulants^8,9^. Poultry diets frequently include oils to improve the concentration of feed energy, boost the availability of fat-soluble vitamins, and improve the palatability of feed all of which enhance feed consumption efficiency. Essential oils promote the synthesis of antioxidant enzymes, improve nutritional digestibility, and improve the intestinal morphology of broilers^10^.

The lemon (*Citrus limon L*.), is a native Asian evergreen plant that is a member of the *Rutaceae* family. Lemon essential oil is well-known for having certain phenolic compounds that have antibacterial, immunomodulatory, antioxidant, and digestive stimulant properties^11^. Previous research showed that including some lemon products in the diets of broilers can enhance overall performance by lowering the detrimental effects of oxidative stress and harmful bacteria^12^. It also contains potassium, calcium, pantothenic acid, thiamine, and beta-carotene, which are compounds that have a number of health benefits. Of the 30 active components found in Lemon essential oil, d-limonene accounted for the largest percentage (47.5%), followed by β-pinene (14.69%), γ-terpinene (9.61%), β-laurene (4.89%), and α-pinene (4.28%)^13^.

A member of the Pedaliaceae family, sesame (*Sesamum indicum L.*) is regarded as a significant dietary supplement in many parts of the world due to its high seed content of protein and oil. Sesame seeds contain bioactive substances including sesamin, sesaminol, and sesamol that have biological roles such as anti-inflammatory, anti-cancer, and antioxidant properties^14^. It is also very nutrient-dense, high in iron, calcium, magnesium, copper, phosphorous acid, silicic acid, and vitamins A, B, and E^15^. Sesame seeds contain a number of bioactive substances, including IP-6 (Phytate), one of the strongest antioxidants yet discovered; lignans, tocopherols, lecithin, pinoresinol, myristic acid, and linoleate, which have been found to be the main antioxidants that give sesame seeds and oil their resistance to oxidative deterioration^16^.

Fenugreek (*Trigonella foenum-graecum L*.) contains bioactive components like alkaloids, saponins, flavonoids, and steroids^17^. It has been shown to have hypoglycemic, hypocholesterolemic, antioxidant, immunomodulatory, digestion-enhancing, anthelmintic, anti-inflammatory, antibacterial, antipyretic, and antimicrobial effects^18–21^. In addition, fenugreek contain of vitamins A, E and D is fenugreek^22^. For broiler chickens, adding fenugreek seeds at a rate of 3g/kg diet as a regular growing booster significantly improves feed consumption, live body weight, and feed conversion ratio^23^. Although numerous studies have investigated the individual properties and bioactivities of essential oils derived from lemon, sesame, and fenugreek, limited attention has been given to the synergistic effects that may arise from combining these oils. This study aims to fill this gap by formulating and assessing a novel mixture of lemon, fenugreek and sesame essential oils, with the hypothesis that their combination may enhance or modify their individual biological effects.

This study was designed to assess the influence of lemon, sesame, fenugreek essential oils and their mixture as feed additive on the growth performance, carcass criteria, nutrient digestibility, cecum microbial and some blood parameters of broiler chickens.

## Materials and methods

### Ethical statement

The experiment was Approved by the Ethics Committee of the Local Experimental Animals Care Committee and performed under the guidelines of the Department of Animal and Poultry production, Faculty of Agriculture, South Valley University, Egypt (Approval code: 1/05/03/25). The study was conducted following ARRIVE guidelines.

### Design, preparation of feed and experimental animals

The Experimental Poultry Farm, Department of Animal and Poultry Production, Faculty of Agriculture, South Valley University, Qena, Egypt, is where this experiment was conducted. The birds were housed in cages made of metabolic wire. Throughout the experiment, the birds had unrestricted access to both water and feed.

Five treatment groups were randomly selected from 240 unsexed broiler chickens (Ross 308 one-day-olds) were procured from a commercial hatchery of Al-Wadi poultry Company. There were six repetitions of each treatment, with eight birds in each. The birds were housed in a typical cage that measured (100 × 60 × 40 cm) and had a 24-h light cycle. The chickens had unrestricted access to diet and water for the duration of the 5-week experiment. According to their treatment, the birds received mash diet. All birds were received starter diet during 1–14 day of age and grower diet during 15–35 day of age. The basal diet was created and prepared to meet the dietary needs NRC^24^. Ingredients and chemical composition of the experimental diets are presented in (Table 1). For 5 weeks, the chicks were provided with their basal diets without supplements (control), supplemented with 400 mg/kg of lemon oil (LEO), supplemented with 400 mg/kg of fenugreek oil (FEO), supplemented with 400 mg/kg of sesame oil (SEO), and supplemented with a 1:1:1 mixture of lemon, fenugreek, and sesame oils (Mix). The LEO, FEO and SEO were purchased from EL Masrayia Company for the extraction of natural oils, Cairo, Egypt.

**Table 1:** Ingredient composition and chemical analysis of the basal experimental diets.

Ingredients (g/100 g)	Starter diet	Grower diet
Maize, ground	27.65	30
Sorghum, ground	27.5	30
Soybean meal (44% CP)	28.55	25
Corn gluten meal (60% CP)	9.55	6
Vit and Min. Premix^a^	0.304	0.3
Sunflower oil	3	5.52
Dicalcium phosphate	2	1.8
Limestone	1	1
Salt	0.304	0.38
DL-methionine	0.042	–
L- lysine HCl	0.1	–
Total	100	100
Chemical analysis		
Dry matter (%)	92.3	92.4
Crude protein (%)	23.26	21.6
Ether extract (%)	5.31	5.75
Crude fibre (%)	2.50	3.78
Ash (%)	6.44	6.18
Calcium (g/kg)	13.22	12.84
Available phosphorus (g/kg)	7.05	7.21
ME k.cal/kg diet	3000	3220

^a^Supplied vitamin-mineral premix contains per kg: 2400.000 IU vitamin A; 1000.000 IU vitamin D; 800 mg vitamin K;16.000 IU vitamin E; 650 mg vitamin B1; 1.600 mg vitamin B2; 1.000 mg vitamin B6; 6 mg vitamin B12; 8.000 mg niacin; 400 mg folic acid; 3.000 mg pantothenic acid; 40 mg biotin; 3.000 mg antioxidant; 80 mg cobalt; 2.000 mg copper; 400 mg iodine; 1.200 mg iron; 18.000 mg manganese; 60 mg selenium; 14.000 mg zinc.

### Growth performance parameters

Throughout the experimental periods, body weight (BW) and feed intake (FI) in each replicates were noted at day 1, 7, 14, 21, 28, and 35 of the experiment. To calculate the feed conversion ratio (FCR) and growth performance (BW gain). Throughout the whole trial period, mortality was documented as it happened. Feed conversion ratio (FCR), was calculated by dividing daily feed consumption by body weight growth.

### Carcass criteria

At the end of experiment 35 days old and had access to water were starved overnight. For each treatment, twenty-four male birds three for each pen were slaughtered by the Islamic method (euthanized by cutting the jugular vein.) and plucked. After removing the head, neck, viscera, shanks, spleen, digestive tract, liver, heart, gizzard, and belly fat, the carcass weight was determined by weighing the remaining body. Each bird’s percentage of live body weight was determined by weighing its liver, heart, empty gizzard, spleen, cecum, and belly fat. The following formula was used to determine the liver percentage:

$${\text{liver}}\;{\text{percentage}}\% = \frac{{{\text{liver}}\;{\text{weight }}}}{{{\text{Live}}\;{\text{body}}\;{\text{weight }}}} \times 100$$

### Digestibility trial and chemical analysis

During the end of the experiment, a total of 60 birds (2 per replicate) were housed in metabolic cages designed for separate housing, which allowed for the accurate collection of complete excreta collection from each bird in isolation. During the digestibility trial, the birds had access to feed and water ad libitum (average daily feed intake was 151 ± 15g/bird). Feces excreta from each individual broiler were collected (130 ± 20 g/bird as wet weight) daily over a continuous 72-h period using trays positioned beneath the cages. To ensure the accuracy of the samples, any feathers and feed particles were carefully removed by hand using forceps and only true excreta were retained for analysis. Feather contamination was minimized through daily cage cleaning. Any feathers found in the collection trays were discarded. The total daily excreta and feed intake per bird were weighed using a digital balance with ± 0.01 g precision. Until it was prepared for chemical analysis, all excreta were stored in a freezer at a steady -20 °C. The excreta had been homogenized before being subjected to chemical analysis. Before being processed into a fine powder using a centrifugal mill and a 1 mm screen, the excrement was further dried in an oven. As stated by AOAC^25^, ether extract was examined using Soxhlet fat analysis (954.02), crude protein by Kjeldahl (984.13), ash by cremation (942.05), and dry matter by oven drying (930.15) for diets and excreta samples. Phosphorous (935.59) and calcium (927.02), and the Parr adiabatic bomb calorimeter (Moline, IL, USA) was used to calculate gross energy for the chemical composition of the feed.

$${\text{Apparent}}\;{\text{digestibility}}\;{\text{of}}\;{\text{protein}},\;{{\% }} = \frac{{\left( {{\text{protein}}\;{\text{ingested}} - {\text{protein}}\;{\text{excreted}}\;{\text{in}}\;{\text{feces}}} \right)}}{{{\text{protein}}\;{\text{ingested }}}} \times 100$$

### Microbiological analysis

At the moment of slaughter, aseptic samples of the cecal were obtained from every selected chicken for microbiological analysis. The cecal was covered with plastic and put in sterile bags with 50 mL of ice-cold cryoprotective broth^26^. and kept until analysis at − 80°^27^. Ceca that had been deep-frozen were thawed for 20 min and taken out of the storage bags for all analytical procedures. After that, the cecal digest’s contents were aseptically transferred into a fresh, sterile bag. It had been homogenized for three minutes after being diluted ten times (10% wt/vol) in sterile, ice-cold anoxic Phosphate-Buffered Saline (PBS 0.1 M, PH 7.0). After that, the digesta slurries were handled as follows. PBS (1 mL) was used to serially dilute each cecal digest homogenate from 10^−1^ to 10^−7^. To *count Escherichia coli* (*E. coli*), *Salmonella* spp, *Lactobacillus* spp, *bacillus* spp and total bacteria, respectively, the bacterial target groups have been identified on duplicate selective agar media M.R.S., MacConkey agar, and *Salmonella* shigella agar following plating dilutions^28^. The plates were incubated for 48 h at 37 °C, and 120 colony-forming units (CFUs) were counted.

### Blood collection and laboratory analysis

Two male birds were selected at random from each replication to serve as representatives at the conclusion of the trial. Using sterilized needles and syringes in vacutainer tubes, blood was extracted from the wing vein in order to get serum. No feed was taken out of the feeder prior to the extraction of blood. The serum was centrifuged for 10 min at room temperature (4000 RPM) after spontaneous separation. For additional examination, serum was gathered in tubes and kept at – 20 °C. Commercial kits (Spectrum Chemical Company, Obour City, Cairo, Egypt) were used to measure the levels of serum total protein, globulin, albumin, glucose, total cholesterol, triglycerides, as well as urea and creatinine for kidney function tests.

### Statistical analysis

The statistical analysis was carried out utilizing a completely randomized design and SAS 9.2’s general linear models (GLM) technique^29^. Pens served as the experimental unit for all analyses. The data were analyzed using one-way ANOVA. We utilized Duncan multiple range tests to compare means. Significance was defined as *P* < 0.05, and a trend toward significance as 0.05 < * P* < 0.10. *P*-values less than 0.001 are denoted as “ < 0.001” instead of the real value.

## Results

### Growth performance

The effect of lemon (LEO), fenugreek (FEO), sesame (SEO) oils, and their combination on growth performance was listed in Table 2. Fenugreek oil supplementation at 400 mg/kg significantly (*P* < 0.05) increased body weight (BW), body weight gain (BWG), and feed intake (FI) during the 1–7, 29–35, and 1–35 day periods and improved feed conversion ratio (FCR) during 1–7 and 15–21 days of age compared to the control group. Dietary including mix of oils significantly (*P* < 0.05) reduced FI as well as improved FCR during the 1–7 day periods compared to the control and LEO groups. During the lengthy trial period, broiler BW, BWG, and FI were unaffected by LEO and SEO supplementation as compared to the control group.

**Table 2:** Effect of Lemon (LEO), fenugreek (FEO) and sesame (SEO) essential oils and their mixture on growth performance of broilers.

Items	Treatments					SEM	P-Value
	C	LEO	FEO	SEO	Mix		
Body weight, g/bird							
1 day	46.20	46.81	46.62	47.36	46.25	0.205	0.385
7 days	225.93^b^	227.41^b^	241.03^a^	226.68^b^	231.58^ab^	1.802	0.032
14 days	595.3	620.7	642.2	579.9	592.6	8.098	0.094
21 days	1084.0	1126.8	1177.8	1051.6	1084.4	15.577	0.085
28 days	1588.7^b^	1621.3^b^	1714.8^a^	1534.1^b^	1590.6^b^	17.066	0.007
35 days	2221.7^b^	2334.1^ab^	2441.7^a^	2198.0^b^	2219.7^b^	25.562	0.004
Body weight gain, g/ bird							
1–7 days	179.72^b^	180.59^b^	194.41^a^	179.31^b^	185.33^ab^	1.759	0.020
8–14 days	369.4	393.3	401.2	353.2	361.0	6.847	0.108
15–21 days	488.7	506.0	535.6	471.7	491.8	9.196	0.251
22–28 days	504.6^ab^	494.5^ab^	536.9^a^	482.5^b^	506.2^ab^	6.540	0.092
29–35 days	633.0^c^	712.8^ab^	726.9^a^	663.8^cb^	629.1^c^	10.864	0.002
1–35 days	2175.5^b^	2287.3^ab^	2395.1^a^	2150.6^b^	2173.5^b^	25.520	0.030
Feed intake g/ bird							
1–7 days	216.1 ^a^	211.9^ab^	211.8^ab^	206.6^b^	208.9^b^	1.020	0.030
8–14 days	428.0	449.1	432.4	409.7	390.2	7.212	0.085
15–21 days	649.6	701.1	675.1	612.4	668.3	10.483	0.082
22–28 days	734.0^ab^	778.3^a^	786.6^a^	711.5^b^	738.2^ab^	8.815	0.022
29–35 days	997.0^cb^	1099.4^ab^	1173.1^a^	1001.9^cb^	970.4^c^	21.551	0.005
1–35 days	3040.9^cb^	3240.0^ab^	3279.2^a^	2942.3^c^	2975.8^c^	41.200	0.012
Feed conversion ratio							
1–7 days	1.2038^a^	1.173^ab^	1.0911^c^	1.152^abc^	1.131^bc^	0.012	0.007
8–14 days	1.157	1.142	1.080	1.158	1.091	0.012	0.141
15–21 days	1.339^ab^	1.385^a^	1.262^c^	1.303^cb^	1.357^ab^	0.016	0.009
22–28 days	1.4593	1.574	1.465	1.477	1.465	0.016	0.135
29–35 days	1.571	1.5423	1.6117	1.5081	1.543	0.013	0.134
1–35 days	1.395	1.416	1.369	1.368	1.369	0.007	0.132

^a,b,c^Means within the same row carrying different superscripts are significantly different at (*P* < 0.05). Control: control, LEO: basal diets supplemented with lemon essential oil at 400 mg/kg, FEO: basal diets supplemented with fenugreek essential oil at 400 mg/kg, SEO: basal diets supplemented with sesame essential oil at 400 mg/kg, Mix: basal diets supplemented with Lemon, fenugreek and sesame essential oils.

### Carcass characteristics

The effect of lemon (LEO), fenugreek (FEO), and sesame (SEO) oils, and their combination on carcass criteria of broilers are present in Table 3. The relative weight of the broiler’s pancreas and gizzard were significantly lower in the dietary regimens containing FEO, LEO, and SEO oils either alone or in combination than in the control group. However, dressing, liver, heart, liver, small intestinal, fat abdominal, cecum weights and small intestine length did not affected by dietary factors.

**Table 3:** Effect of Lemon (LEO), fenugreek (FEO) and sesame (SEO) essential oils and their mixture on carcass criteria of broilers.

Items	Treatments					SEM	*P*-value
	C	LEO	FEO	SEO	Mix		
LBW	2231.7	2388.3	2465.8	2210.8	2082.5	48.365	0.083
Dressing %	75.294	76.083	75.097	76.751	75.617	0.798	0.973
Liver %	1.933	2.106	1.910	1.849	2.072	0.077	0.827
Gizzard %	1.722^a^	1.235^b^	1.094^b^	1.334^b^	1.389^b^	0.057	0.003
Heart %	0.489	0.527	0.469	0.531	0.540	0.016	0.639
Pancrease %	0.240^a^	0.135^b^	0.1359^b^	0.169^b^	0.132^b^	0.010	0.001
Small intestinal weight %	4.462	4.214	4.661	4.301	3.652	0.198	0.598
Small intestinal L (cm)	184.5	180.0	195.8	181.6	176.6	3.229	0.408
Fat abdominal%	0.327	0.267	0.242	0.291	0.274	0.009	0.626
Cecum weight %	0.607	0.866	0.695	0.774	0.734	0.029	0.057

^a,b,c^Means within the same row carrying different superscripts are significantly different at (*P* < 0.05). Control: control, LEO: basal diets supplemented with lemon essential oil at 400 mg/kg, FEO: basal diets supplemented with fenugreek essential oil at 400 mg/kg, SEO: basal diets supplemented with sesame essential oil at 400 mg/kg, Mix: basal diets supplemented with Lemon, fenugreek and sesame essential oils.

### Nutrient digestibility

**Fig. 1:**
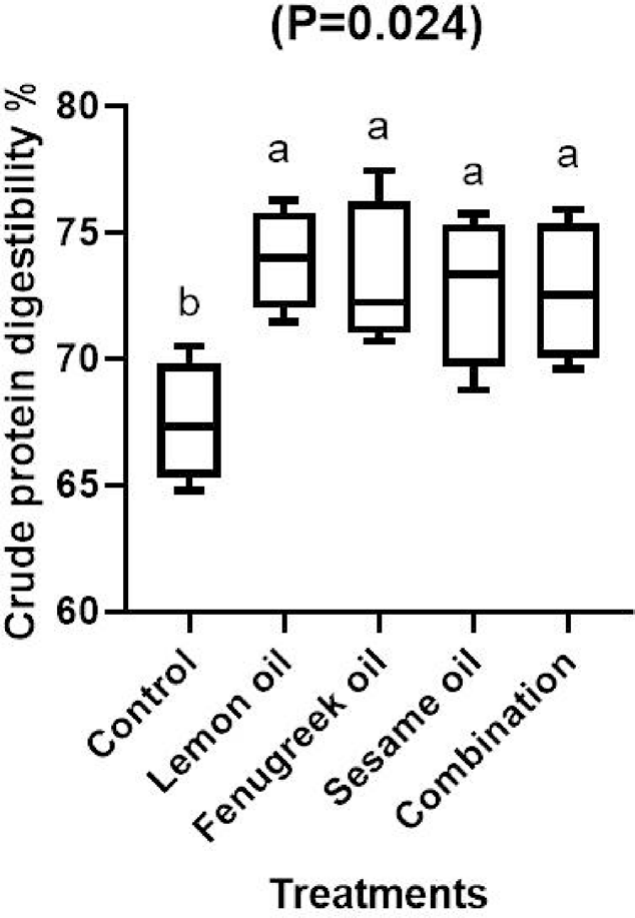
The effect of dietary including lemon (LEO), fenugreek (FEO), and sesame (SEO) oils, and their combination on crude protein digestibility of broilers.

**Fig. 2:**
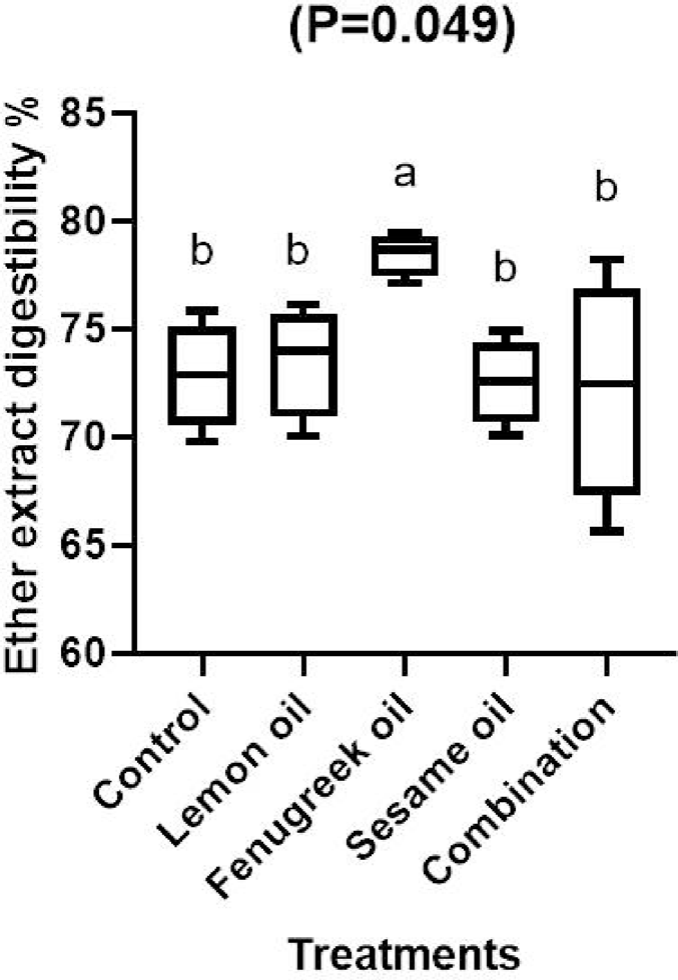
The effect of dietary including lemon (LEO), fenugreek (FEO), and sesame (SEO) oils, and their combination on ether extract digestibility of broilers.

**Fig. 3:**
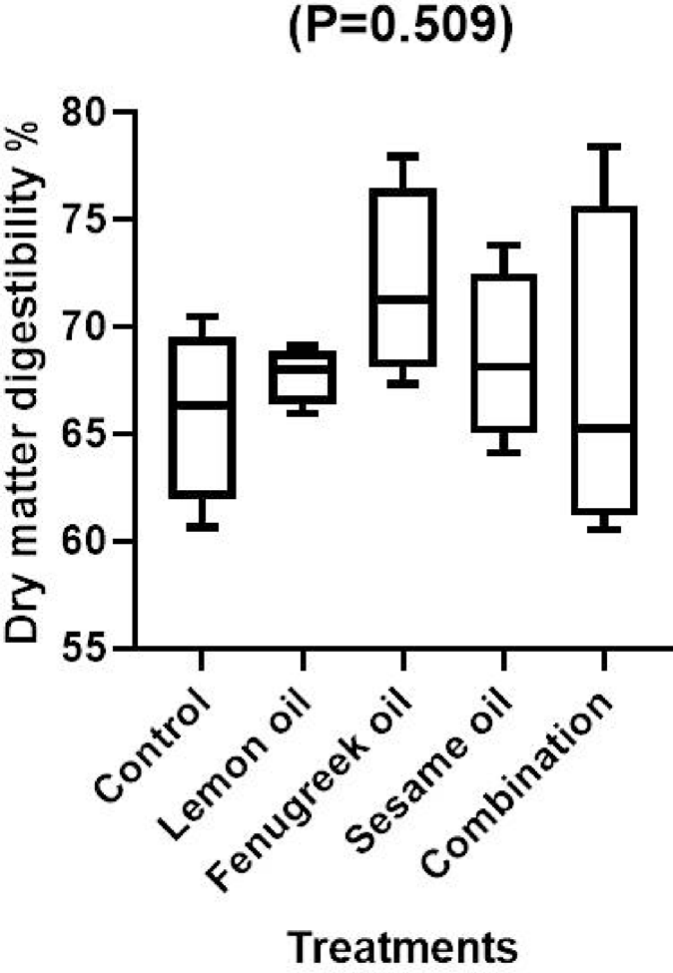
The effect of dietary including lemon (LEO), fenugreek (FEO), and sesame (SEO) oils, and their combination on dry matter digestibility of broilers.

Crude protein digestibility was significantly (*P* < 0.05) higher in broilers fed a diet supplemented with LEO, FEO, SEO either separately or in combination than the control group (Fig. 1). Additionally, broilers fed diet supplemented with FEO 400 mg/kg significantly (*P* < 0.05) improved digestibility of ether extract compared to other treatments (Fig. 2). However, digestibility of dry matter did not affected by dietary factors (Fig. 3).

### Cecal microbiota

Effect of dietary lemon (LEO), fenugreek (FEO), and sesame (SEO) oils, and their combination on the cecal microbiota of broilers are present in Table 4. When compared to the control and Mix groups, the addition of 400 mg/kg of LEO to the broiler feed significantly (*P* < 0.01) decreased the overall counts of bacteria, *E. coli, Salmonella*, and *Bacillus*. Additionally, According to the current findings, broilers provided a diet supplemented with 400 mg/kg of FEO had considerably (*P* < 0.01) lower *Salmonella* counts than the control, SEO, and Mix groups. Additionally, the counts of *Lactobacillus* were higher than those of the control group. In contrast to the control and FEO groups, the addition of 400 mg/kg SEO and Mix oils resulted in a decrease in the overall bacterial count and an increase in *Lactobacillus* compared to the control group.

**Table 4:** Effect of Lemon (LEO), fenugreek (FEO) and sesame (SEO) essential oils and their mixture on cecal microbiota (log 10 CFUs) of broilers.

Items	Treatments					SEM	*P*-value
	C	LEO	FEO	SEO	Mix		
Total count	9.777^a^	9.312^b^	9.973^a^	9.502^b^	9.469^b^	0.056	0.001
*E. coli count*	4.617^a^	3.879^b^	4.807^a^	4.835^a^	4.845^a^	0.081	0.001
*Salmonella count*	4.762^a^	3.607^b^	3.962^b^	4.993^a^	4.891^a^	0.135	0.001
*Lactobacillus*	7.529^c^	7.472^c^	7.890^b^	8.155^a^	7.835^b^	0.056	0.001
*Bacillus*	6.730^ab^	5.772^c^	6.931^a^	6.851^ab^	6.663^b^	0.092	0.001

^a,b,c^Means within the same row carrying different superscripts are significantly different at (*P* < 0.05). Control: control, LEO: basal diets supplemented with lemon essential oil at 400 mg/kg, FEO: basal diets supplemented with fenugreek essential oil at 400 mg/kg, SEO: basal diets supplemented with sesame essential oil at 400 mg/kg, Mix: basal diets supplemented with Lemon, fenugreek and sesame essential oils.

## Blood biochemistry

### Total protein and albumin

**Fig. 4:**
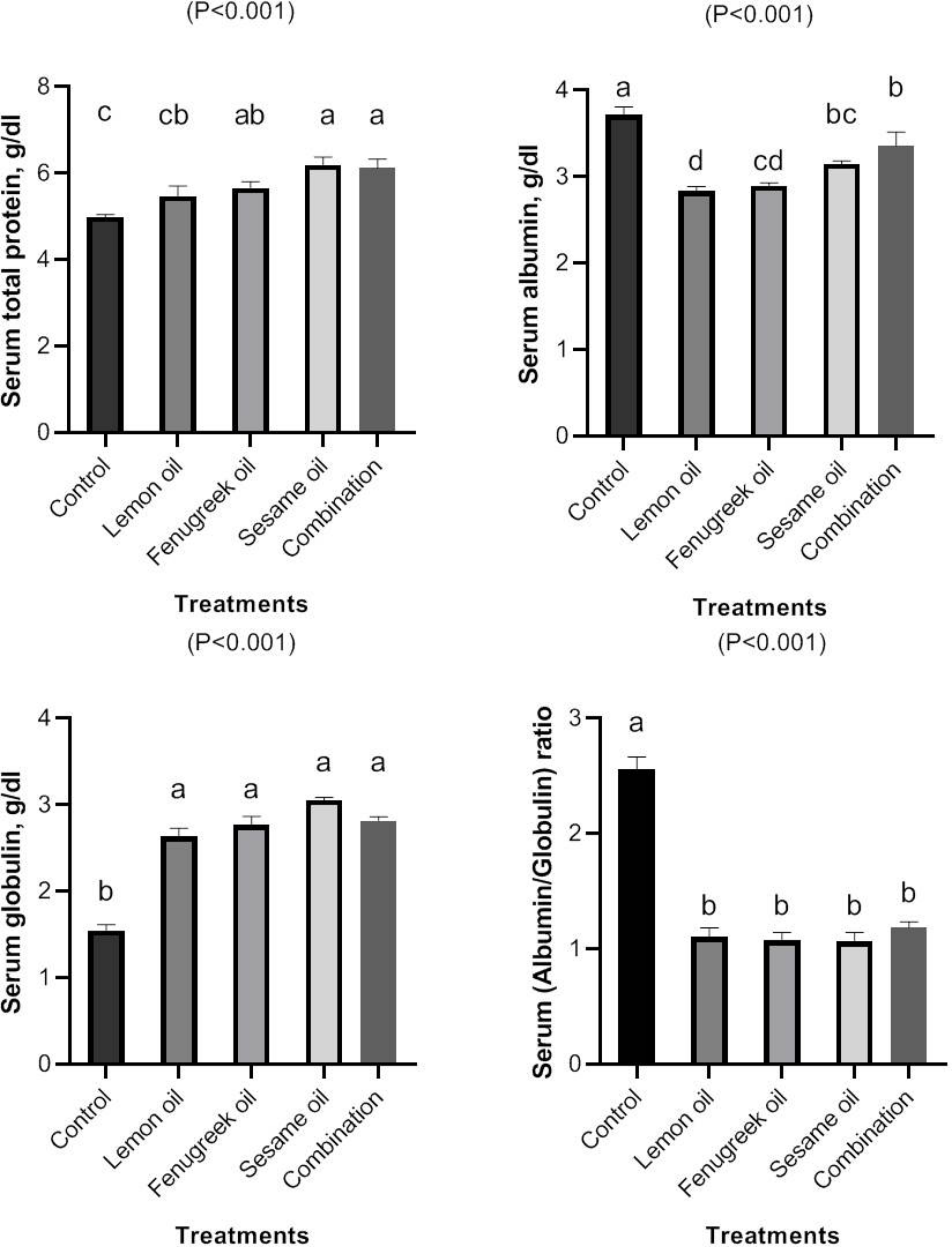
The effect of dietary including lemon (LEO), fenugreek (FEO), and sesame (SEO) oils, and their combination on serum concentrations of total protein, albumin, globulin and A/G ratio.

The results of serum measurements of total protein, albumin, globulin and A/G ratio concentrations following the feeding of broiler chick’s lemon (LEO), fenugreek (FEO), and sesame (SEO) oils, either separately or in combination are present in (Fig. 4). Datary containing 400 mg/kg FEO, LEO, and SEO either alone or in combination significantly (*P* < 0.01) improved serum levels of total protein and globulin while decreasing serum levels of albumin and A/G ratio in comparison to the control group. Serum concentration of albumin was lower in broilers fed diet supplemented with LEO and FEO than SEO and combination group.

### Kidney function

**Fig. 5:**
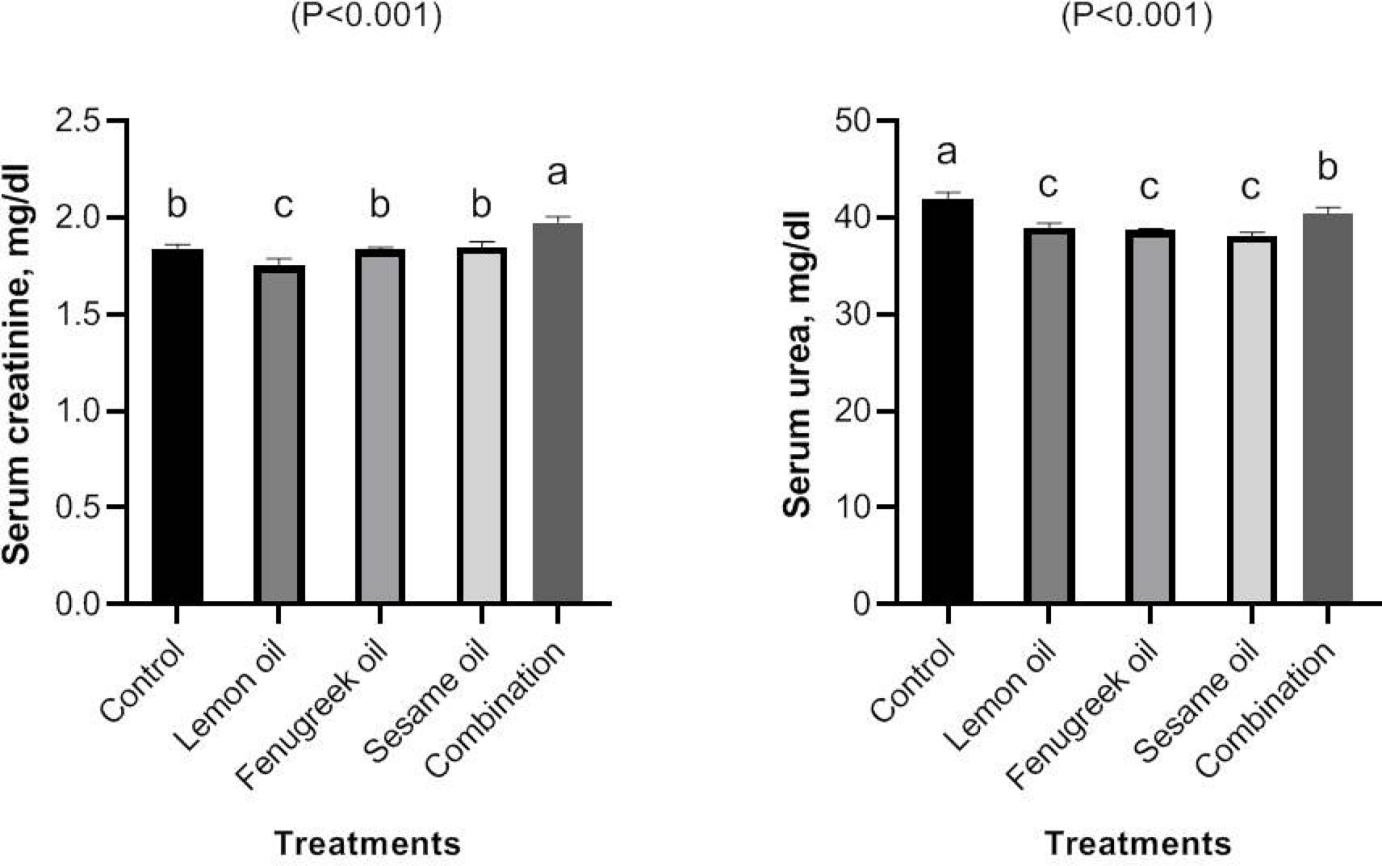
The effect of dietary including lemon (LEO), fenugreek (FEO), and sesame (SEO) oils, and their combination on serum concentrations of creatinie and urea.

The results of measuring the serum concentrations of creatinine and urea after feeding broilers lemon (LEO), fenugreek (FEO), and sesame (SEO) oils, and their combination are displayed in (Fig. 5). Supplementation of FEO, LEO, and SEO either alone or in combination significantly (*P* < 0.01) decreased serum levels of urea compared to the control group. Serum concentration of creatinine was increased in broilers received 400 mg/kg Mixture oils compared to the control, LEO, SEO and FEO groups, the lower value of creatinine was observed in LEO group (*P* < 0.01).

### Glucose

**Fig. 6:**
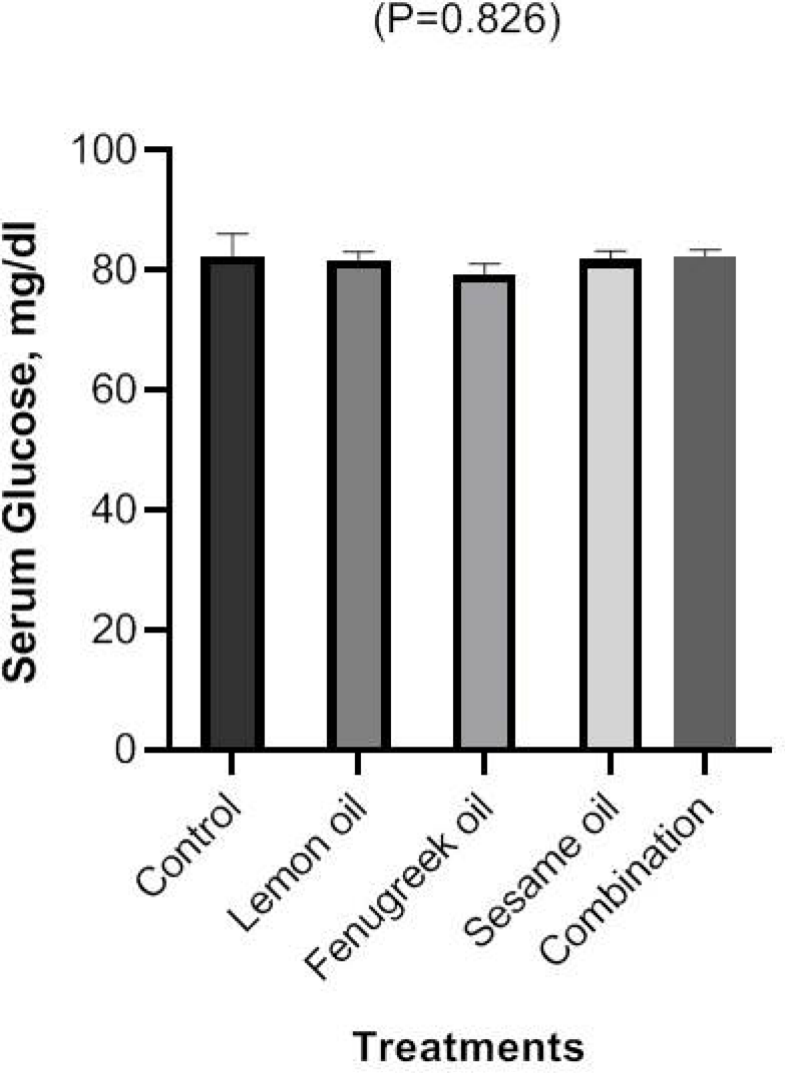
The effect of dietary including lemon (LEO), fenugreek (FEO), and sesame (SEO) oils, and their combination on serum concentrations of glucose.

Figure 6 shows the results of measuring the serum concentrations of glucose after broilers were fed lemon (LEO), fenugreek (FEO), and sesame (SEO) oils, and their combination. There were non-significantly changes in serum concentration of glucose of broilers fed FEO, LEO, and SEO either alone or in combination.

### Lipid profile

**Fig. 7:**
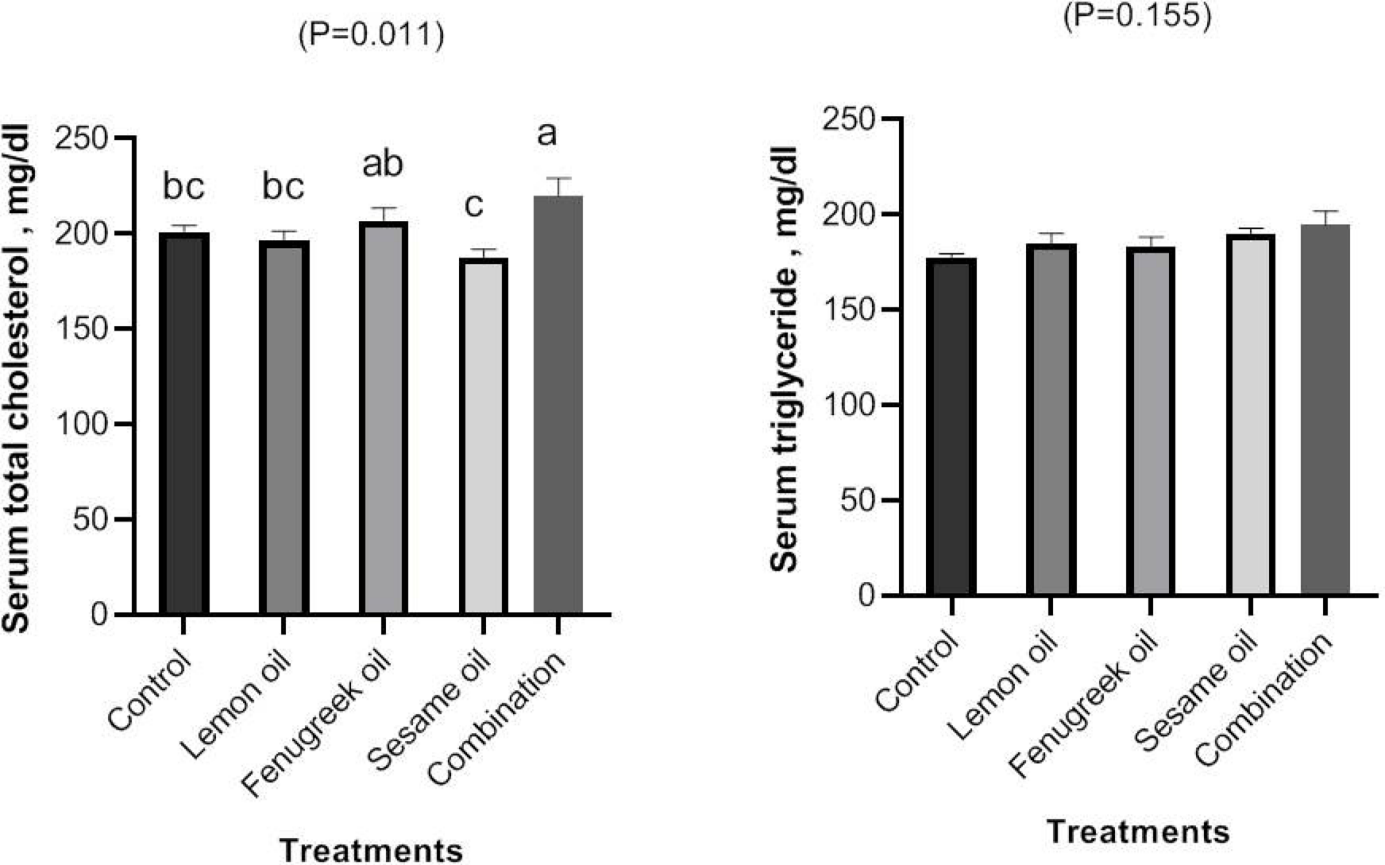
The effect of dietary including lemon (LEO), fenugreek (FEO), and sesame (SEO) oils, and their combination on serum concentrations of total cholesterol and triglycerides.

The effect of dietary including lemon (LEO), fenugreek (FEO), and sesame (SEO) oils, and their combination on serum concentrations of total cholesterol and triglycerides of broilers are present in Fig. 7. Broilers fed a diet supplemented with mixture oils had higher serum concentrations of total cholesterol than other groups, the SEO group had the lowest concentration of total cholesterol (*P* < 0.01). Triglyceride levels in the serum also showed non-significant alterations.

## Discussion

Throughout the experiment, there was no mortality. In the current study, Fenugreek oil supplementation at 400 mg/kg significantly (*P* < 0.05) increased body weight (BW), body weight gain (BWG), and feed intake (FI) during the 1–7, 29–35, and 1–35 day periods and improved feed conversion ratio (FCR) during 1–7 and 15–21 days of age compared to the control group. Additionally, Dietary including mix of oils significantly (*P* < 0.05) reduced FI as well as improved FCR during the 1–7 day periods compared to the control and LEO groups. These results imply that fenugreek oil improves broiler performance, which may be explained by its bioactive constituents, which include alkaloids, flavonoids, and saponins that are known to improve digestion and growth. Which stimulate the digestive system, increase the consumption of feed, and activate the hypothalamus gland, in addition to the essential fatty acids and high-quality proteins found in fenugreek^30,31^. According to Amein et al.^32^, adding fenugreek to broiler diets may improve biological and metabolic functions, maximize nutrient use, and raise feed conversion rates. These outcomes agree with the findings of Yang et al.^33^, who indicated that broiler BW, BWG, and FCR were considerably improved by nutritional supplementation of fenugreek seed extracts at 50 and 100 mg/kg compared to the control group. Additionally, broilers fed diets supplemented with 0.5% fenugreek showed significant (*P* < 0.05) improvements in BW, BWG, and FCR^34,35^. Fenugreek oil impact on feed consumption might be explained by a variety of viewpoints, including the idea that it is a natural feed additive that improves diet palatability and increases feed intake. This may be connected to the development of the broiler chicks’ gut morphological changes, which can be caused by variations in the gut fluid’s microbial content, including its metabolites^23^.

In the current finding, the relative weight of the broiler’s pancreas and gizzard were significantly lower in the dietary regimens containing LEO, FEO, and SEO oils either alone or in combination than in the control group. These outcomes agree with the findings of Parvizi et al.^36^ who observed a significant (*P* < 0.05) reduced in the relative weigh of gizzard in broilers fed a diet supplemented with 300 ppm of FEO compared to the control group. Likewise, during the finisher phase dietary including 2 and 3 g/kg fenugreek seed powder significantly (*P* < 0.05) lower gizzard weight of broilers than the control group^37^.

The current results show that broilers given a diet supplemented with LEO, FEO, and SEO either independently or in combination had considerably (*P* < 0.05) higher crude protein digestibility than the control group (Fig. 1). Furthermore, when compared to other treatments, the digestibility of ether extract was significantly (*P* < 0.05) improved in broilers fed a diet supplemented with 400 mg/kg of FEO (Fig. 2). The findings imply that these essential oils might improve broiler growth and feed efficiency by having positive impacts on nutrient use. The lipophilic nature of the components in fenugreek oil, which improve lipid metabolism and absorption, may be the reason for the notable improvement in ether extract digestibility observed in the group supplemented with 400 mg/kg of FEO. According to earlier research, fenugreek-derived flavonoids and saponins can improve lipid digestibility by promoting bile secretion, emulsification, and micelle formation^38^. The higher EE digestibility in broilers fed FEO when compared to other treatments may be explained by this mechanism. Numerous studies demonstrated that adding essential oils improved the intestinal microbial ecosystem’s balance and increased the activity of digestive enzymes, both of which improved nutritional digestion^39,40^. Other finding has demonstrated that adding essential oils did not improve the digestion of nutrients^41,42^. The variation in outcomes could be caused by the kinds of essential oils used or the level used, since certain oils have a negative (irritating) effect on the intestinal lining wall, which causes mild inflammation^43^, and lowers nutritional utilization. Similar results was observed by Hafeez et al.^38^ who found that mixture of fenugreek, coriander and garlic at a level of 1% significantly improved digestibility of ether extract and crude protein compared to the control group. Our results are consistent with those of Elbaz et al.^44^ who found that broilers feeding a diet supplemented with LEO at 200 mg/kg either alone or in conjunction with garlic oil had significantly (*P* < 0.01) better digestibility of dry matter and crude protein when compared to the control group.

Numerous intestinal microorganisms found in the digestive system are crucial to the digestion, absorption, and general health of the bird. *Salmonella* spp. and *Escherichia coli* (E. coli) are prevalent pathogenic “Gram-negative bacteria” that limit broiler growth rate and disturb the gut microbiota^45,46^. Feed additives have an essential role in altering the intestinal bacteria composition. In the current findings, broilers provided a diet supplemented with 400 mg/kg of FEO had considerably (*P* < 0.01) lower *Salmonella* counts than the control, SEO, and Mix groups. Additionally, the counts of Lactobacillus were higher than those of the control group. Salmonella species are important foodborne pathogens that affect chicken productivity and can be harmful to human health^47^. Fenugreek’s antibacterial qualities, which include bioactive substances including alkaloids, flavonoids, and saponins, are probably responsible for the observed decrease in Salmonella counts in broilers treated with FEO. These outcomes agree with the findings of Parvizi et al.^36^ who observed a significant (*P* < 0.05) increased the counts of Lactobacillus in broilers fed diet supplemented with 100, 200 and 300 ppm of FEO compared to the control group. In the current finding, the addition of 400 mg/kg of LEO, and SEO to the broiler feed significantly (*P* < 0.01) decreased the overall counts of bacteria, *E. coli, Salmonella, Coliform and Bacillus*. Liner and quadratic decreased in counts of bacteria, *E. coli, Salmonella* as well as increased (*P* < 0.001) *Lactobacillus* of quails fed diet supplemented with lemongrass essential oil at 150, 300, 450, and 600 mg/kg compared to the control group^8^. It is well established that essential oils have antibacterial, gut microbiota-balancing, and digestive enzyme-enhancing affects that increase nutritional absorption and digestion. However, when broiler diets were supplemented with 200 mg/kg of garlic oil and lemon oil, either alone or in combination, the cecal *E. coli* and *Lactobacillus* counts were significantly higher (*P* < 0.05) than those of the control groups^44^.

Blood biochemical profiles can represent a variety of physiological changes in animals, including those associated with age, species, season, nutrition and physiological condition^48^. Among the numerous vital roles that serum total proteins play in the body are energy production, muscle repair, and immune system support. According to the current findings, datary containing 400 mg/kg FEO, LEO, and SEO either alone or in combination significantly (*P* < 0.01) improved serum levels of total protein and globulin while decreasing serum levels of albumin and A/G ratio in comparison to the control group. During the finisher phase dietary including 3 g/kg fenugreek seed powder significantly (*P* < 0.05) decreased serum levels of albumin of broilers compared to the control group^37^. Serum total protein and albumin were quadratic increased (*P* < 0.05) when quails fed a diet supplemented with lemongrass essential oil at 150, 300, 450, and 600 mg/kg compared to the control group^8^. However, Elbaz et al.^44^ found that supplied LEO at 200 mg/kg, either by alone or in conjunction with garlic oil, had no effect on the broiler’s serum total protein, globulin, albumin, A/G ratio, creatinine and urea.

Two essential indicators for assessing kidney function are serum creatinine and urea. According to the current study, supplementation of LEO, FEO, and SEO either alone or in combination significantly (*P* < 0.01) decreased serum levels of urea compared to the control group. Additionally the lower value of serum creatinine was observed in LEO group (*P* < 0.01) than other groups. The active component of essential oils resalable for improved the kidney functions of broilers.

In the current study, Broilers fed a diet supplemented with mixture oils had higher serum concentrations of total cholesterol than other groups, the SEO group had the lowest concentration of total cholesterol (*P* < 0.01). Dietary including 2.5 and 3% fenugreek seed powder significantly (*P* < 0.05) lower serum concentration of total cholesterol of broilers than the control group^49^. Liner and quadratic decreased in serum concentrate of total cholesterol, triglyceride of quails fed diet supplemented with 300, 450, and 600 mg/kg compared to the control group^8^. Serum concentration of total lipids and triglycerides of Japanese quail fed dietary supplemented with 2 and 4% SEO were lower (*P* < 0.05) than the control group^50^. Vitamin E and selenium, two potent antioxidants that can prevent heart disease, are found in sesame seeds or oil^50^. Sesame improves vitamin E’s antioxidant activity and sesamolin inhibits lipid peroxidation^51^. Supplementing vegetable oils high in linolenic acid and linoleic to the quail diet improves physiological indicators and increases economic efficiency without negatively affecting performance^52^. Therefore, the findings of this study could indicate that microbial balance, nutrient digestibility, performance, general health condition of broilers were enhanced by feeding chick’s lemon, fenugreek, and sesame oils, either independently or in combination.

## Conclusion

The present results indicated that supplemented lemon, fenugreek, and sesame oils, and their combination at 400 mg/kg as a feed additives improved microbial balance, nutritional digestibility, and overall health status of broilers chicks during the period from 1- 35 days of age. Among the evaluated treatment broilers chicks fed 400 mg/kg of fenugreek oil showed better growth performance, and overall health status than other treatments. Further research is warranted to elucidate the exact mechanisms underlying these effects and to explore the potential synergistic interactions among these essential oils.

## Data Availability

The datasets generated and/or analyzed during the current study are available from the corresponding author on reasonable request.

## Abbreviations

A/G ratio: Albumin/globulin ratio
AOAC: Association of Official Analytical Chemists
BW: Body weight
BWG: Body weight gain
CP: Crude protein
*E. Coli*: *Escherichia coli*
FCR: Feed conversion ratio
FEO: Fenugreek essential oil
FI: Feed intake
LEO: Lemon essential oil
SEO: Sesame essential oil
